# The suicide prevention effect of lithium: more than 20 years of evidence—a narrative review

**DOI:** 10.1186/s40345-015-0032-2

**Published:** 2015-07-18

**Authors:** U Lewitzka, E Severus, R Bauer, P Ritter, B Müller-Oerlinghausen, M Bauer

**Affiliations:** Department of Psychiatry and Psychotherapy, Universitätsklinikum Carl Gustav Carus Dresden, Technische Universität Dresden, Fetscherstr. 74, 01307 Dresden, Germany; Drug Commission of the German Medical Association, Berlin, 10623 Germany

**Keywords:** Lithium, Suicide, Suicide attempt, Mortality, Depression, Prevention, Affective disorders, Bipolar disorders

## Abstract

The management and treatment of patients with suicidal behavior is one of the most challenging tasks for health-care professionals. Patients with affective disorders are at high risk for suicidal behavior, therefore, should be a target for prevention. Numerous international studies of lithium use have documented anti-suicidal effects since the 1970s. Despite the unambiguous evidence of lithium’s anti-suicidal effects and recommendations in national and international guidelines for its use in acute and maintenance therapy of affective disorders, the use of lithium is still underrepresented. The following article provides a comprehensive review of studies investigating the anti-suicidal effect of lithium in patients with affective disorders.

## Review

### Introduction

Completed and attempted suicides and suicidal thoughts represent complex phenomenon with changing definitions (De Leo et al. 2006). Completed and attempted suicide can cause immense individual and familial distress. Suicide is among the top 20 leading causes of death globally among all ages. Every year, nearly one million people die from suicide (WHO 2014), including approximately 58,000 within the European Union and 10,000 in Germany. In individuals aged 15–39 years, suicide is the second leading cause of death after accidents; however, the current number of suicide attempts remains unclear. The ratio of suicide attempts to death by suicide in youth is estimated to be approximately 25:1, compared to approximately 4:1 in the elderly (AFSP 2014).

The etiology of suicidal behavior and suicidal ideation is multi-factorial, although one of the most common risk factors is having a psychiatric disease (Lee and Kim 2010). Several psychological autopsy studies have supported high rates of psychiatric disorders among individuals who die by suicide (Cavanagh et al. 2003). Further, a meta-analysis of 3275 suicides reported that 87.3 % of suicide completers had been diagnosed with a psychiatric disorder prior to the suicide (Arsenault-Lapierre et al. 2004).

It is estimated that the lifetime risk of suicide among individuals with mood disorders (particularly major depression) ranges from 6 to 15 % (WHO 2000). Nordentoft et al. (2011) showed that among Danish men, followed for 36 years, the absolute risk of completed suicide was highest for those with bipolar disorder followed by unipolar affective disorder.

Findings from a meta-analysis indicate that the strongest risk factors for suicide in patients suffering from bipolar disorder are a previous suicide attempt and hopelessness. A positive family history of suicide, early onset of bipolar disorder, rapid cycling, and drug/alcohol dependency were identified as risk factors for non-fatal suicidal behavior (Hawton et al. 2005). Recently, the ISBD Task Force (Schaffer et al. 2015) published a meta-analysis indicating that female gender, younger age at illness onset, and comorbid anxiety disorder are associated with suicide attempts in bipolar patients while male gender and a family history of suicide in a first-degree relative is associated with suicide deaths.

Differences in suicide attempts have been reported in patients with bipolar disorder I compared to bipolar disorder II (Novick et al. 2010). Specifically, patients with bipolar disorder (BD) II use significantly more violent and lethal methods compared to individuals with BD I.

Treatment of patients with suicidal behavior is one of the most challenging tasks for health-care professionals. Due to the high mortality, morbidity, and costs related to attempted suicide, the development of treatment and prevention strategies for suicidal behavior have been the focus for much of the research on suicidality. Since the early 1970s, several international studies confirmed the anti-suicidal effect of lithium. Despite convergent evidence and corresponding recommendations in the national and international guidelines for the use of lithium for acute and maintenance therapy of affective disorders (Pfennig and Bauer 2013; Grunze et al. 2013), the use of lithium is still uncommon.

The study of suicide is methodologically challenging. Firstly, owing to ethical concerns, when patients develop suicidal behavior in a study, they are often required to drop-out, particularly in pharmaceutical studies. Moreover, suicidal behavior and thoughts are often an exclusion criterion. Ethically, it is not acceptable to design a randomized controlled study with completed suicide or suicide attempts as the primary outcome within a placebo-controlled arm—therefore, most often a “treatment as usual” condition is a mandatory component of the protocol. Secondly, completed suicide and suicide attempts are rare events. To achieve sufficient statistical power, a large number of patients are required or the study observation period needs to be long, and both of these scenarios can be costly.

The following review provides a chronological overview of randomized controlled trials (RCT) and follow-up studies on patients from specialized lithium clinics as well as epidemiological studies using reported data from registers or health insurance claims. We also focus on reviews and meta-analyses investigating the anti-suicidal effect of lithium in patients with affective disorders as a primary goal or within a retrospective secondary analysis. Despite the limitations in data quality, we also included early case reports and clinical observations to add clinical context. Clinical implications for the treatment of affective disorders are discussed.

### Methods

A comprehensive literature review was conducted using the online search engine MEDLINE, focusing on clinical trials and cross-sectional and longitudinal studies between 1970 and 2014. The search terms used were: “suicidal ideation,” “suicide,” “suicide attempt,” “suicidal behavior,” “affective disorder,” “lithium,” “depression,” “depressive disorder,” “bipolar disorder,” and “major depression.” Two researchers (UL and ES) identified all trials that examined the treatment effects of lithium with or without a comparison with other substances (i.e., mood stabilizer, antidepressants, and antipsychotics) in patients with affective disorders and presented results on suicidal behavior. Historically, the anti-suicidal properties of lithium were first described in case reports and small studies. Therefore, some of these earlier reports published after 1970 were included in this review.

Most studies measured the general prophylactic efficacy of lithium in the treatment of affective episodes while death due to suicide, suicide attempts, or deaths from all causes are reported as secondary outcomes. Further, several studies investigating lithium in the treatment of affective disorders did not typically report mortality data. Therefore, no conclusion can be drawn about lithium’s anti-suicidal effect in these specific studies. So far, only one double-blind, placebo-controlled trial examined the occurrence of completed suicide and suicide attempts as a primary outcome in patients treated with lithium.

### Results

Our search yielded 339 potentially relevant studies. After inspection of all abstracts, 20 randomized controlled trials (Table 1) and 28 follow-up and epidemiologic studies (Table 2) focusing on attempted and completed suicides, suicidal ideation, or mortality (long-term follow-up of lithium-treated patients and epidemiological studies including record linkage) were included in the review. We also included five studies (Table 3) on the potential anti-suicidal effects of lithium as a trace element in drinking water as well as four meta-analyses (Table 4).

**Table 1:** Randomized controlled trials—not specifically focusing on suicidality or mortality

Year/country	Author	Diagnoses	Medication/type of study	Number of patients	Duration	Results
1971/UK	Coppen et al.	Unipolar	Li	65	2 years	3 died (Plac) from causes not related to affective disorders
		Bipolar	Placebo	Li (*n* = 28)		
				Plac (*n* = 37)		
			Randomly assigned	Unipolar Li (*n* = 11) Unipolar Plac (*n* = 17) Bipolar Li (*n* = 15) Bipolar Plac (*n* = 22)		
1973/USA	Prient et al. (a)	Unipolar	Li	122	2 years, observation period started after patient discharge	1 suicide (Plac), 4 deaths due to other causes not related to affective disorder
		Bipolar	Imipramine	Li (*n* = 45) Imipramine (*n* = 38) Plac (*n* = 39)		
			Placebo			
			Randomly assigned	Bipolar (*n* = 44)		
				Unipolar (*n* = 78)		
1973/USA	Prien et al. (b)	Bipolar	Li	205	2 years	1 suicide (Plac)
			Placebo	Li (*n* = 101)		2 deaths due to other causes
				Plac (*n* = 104)		
			Randomly assigned			
1989/USA	Dorus et al.	Depressed vs. non-depressed alcoholics	Li compliant	457	52 weeks	1 death from all causes (Plac), no death (Li)
			(*n* = 82)	Depressed (*n* = 171 ≥ 108 study completers)		
			Placebo compliant (*n* = 82)			
				Non-depressed (*n* = 286 ≥ 172 study completers)		
			Double-blind, placebo-controlled trial			
1996/Germany	Greil et al.	Unipolar depression	Li	81	2.5 years	1 suicide (amitriptyline) 0 suicide (Li)
			Amitriptyline	Li (*n* = 40)		
			Prospective randomized multicenter trial	Amitriptyline (*n* = 41)		
1997/Germany	Greil et al. (a)	Bipolar disorder	Li	144	2.5 years	0 suicide or SA (Li)
			CBZ	Li (*n* = 74)		1 suicide, 1 SA (CBZ)
				CBZ (*n* = 70)		
			Prospective randomized multicenter trial			
1997/Germany	Greil et al. (b)	Schizoaffective disorder	Li	90	2.5 years	0 suicide or SA (Li)
			CBZ	Li (*n* = 43)		0 suicide, 4 SA (CBZ)
				CBZ (*n* = 47)		
			Prospective randomized multicenter trial			
2000/UK	Coppen et al.	Unipolar	Li	103	Study population was recruited in 1977	10 patients died during the study, expected number of deaths was 18.31 and no deaths from suicide
		Bipolar	Placebo	Unipolar (*n* = 67) Bipolar (*n* = 30) Schizoaffective (*n* = 6)		
		Schizoaffective	Randomly assigned			
2000/Germany	Bauer et al.	Refractory depression	AD + Li	30	4.5 months	1 suicide (Plac)
			AD + placebo	AD + Li (*n* = 14)		
				AD + Plac (*n* = 15)		
			Randomized, double-blind, placebo-controlled trial			
2002/UK	Wilkinson et al.	Unipolar depression	AD + Li	49	2 years	4 deaths from unrelated causes (2 Li, 2 Plac)
			AD + placebo	AD + Li (*n* = 25)		
				AD + Plac (*n* = 24)		
			Randomized, double-blind, controlled trial	Elderly patients (>65)		
2003/Europe, Canada, USA	Calabrese et al.	Bipolar I	Li	966	8–16 weeks open label, 18 months	6 deaths: 4 suicides, 2 of the suicides during open-label study, 1 suicide after discontinuation from open-label study
			Placebo	Li (*n* = 121)		
			LTG	Plac (*n* = 121)		
				LTG (*n* = 221)		
			Randomized, double-blind, placebo-controlled trial			
						0 suicide (Li), 1 suicide (LTG)
						11 SA (10 open label, 1 in Plac)
2005/USA	Findling et al.	Bipolar I, II	Li	60	Phase 1: up to 20 weeks	0 suicide, 0 SA in both groups
			VLP	Li (*n* = 30)		
				VLP (*n* = 30)	Phase 2: 76 weeks	
			Randomized, double-blind, controlled trial	Pediatric (5–17 years)		
2005/USA, Africa, Australia, Canada, Europe	Tohen et al.	Bipolar I	Li	431	Open label with Li + OLZ 6–12 weeks, 52 weeks double-blind phase monotherapy	1 suicide (Li) during open-label phase, 2 died (Li) during double-blind phase (1 suicide, 1 accident)
			OLZ	Li (*n* = 214)		
				OLZ (*n* = 217)		
			Randomized, double-blind, controlled trial			
2007/Netherlands	Kok et al.	Treatment resistant depression	Augmentation with Li	29	6 weeks + 2 years follow-up	2 deaths (Li), 3 deaths (phenelzine)
				Li (*n* = 15)		
			Augmentation with phenelzine	Phenelzine (*n* = 14)		
				Elderly (>60 years)		
			Open, randomized controlled trial			
2008/Germany	Lauterbach et al.	Depressive disorders	Li	167	12 months	0 suicide (Li)
			Placebo	Li (*n* = 84)		3 suicides (Plac)
				Plac (*n* = 83)		7 SA (Li)
			Randomized, double-blind, placebo-controlled trial			7 SA (Plac)
2010/UK, France, USA, Italy	Geddes et al.	Bipolar I	Li	330	24 months	2 deaths from all causes (Li), 3 deaths from all causes (VLP), 1 death from all causes (Li + VLP)
			VLP	Li (*n* = 110)		
			Li + VLP	VLP (*n* = 110)		
				Li + VLP (*n* = 110)		
			Randomized, open-label trial			
2010/Denmark, Sweden	Licht et al.	Bipolar I	Li	155	52 weeks	1 suicide (Li)
			LTG	Li (*n* = 78)		1 SA (LTG)
				LTG (*n* = 77)		
			Open, randomized trial			
2011/USA	Oquendo et al.	Bipolar disorder	Li	98	2.5 years	0 suicide
			VLP	Li (*n* = 49)		6 SA (Li), 8 SA (VLP)
			Randomized, double-blind controlled trial	VLP (*n* = 49)		45 suicidal events (16 Li, 18 VLP)
2011/Asia, Europe, Central and South America, USA	Weisler et al.	Bipolar I	Li	2438 (open label)	Open label 4–24 weeks, double-blind up to 104 weeks	3 deaths (Quet): 2 due to other causes, 1 suicide/accidental gunshot, similar overall incidence of suicidal behavior/ideation in the Quet (*n* = 3), Li (*n* = 3), and Plac (*n* = 8) groups
			Quetiapin	1226 (double-blind)		
			Placebo	Li (*n* = 364)		
				Quet (*n* = 404)		
			Randomized, placebo-controlled trial	Plac (*n* = 404)		
2011/USA	Khan et al.	Depressive disorders	Cit + Li	80	4 weeks	Subgroup of the patients assigned to citalopram and lithium had significantly higher Sheehan Suicidality Tracking Scale (S-STS) remission rates compared to patients assigned to citalopram and placebo
			Cit + Placebo	Cit + Li (*n* = 40)		
				Cit + Plac (*n* = 40)		
			Randomized, double-blind trial			

*SA* suicide attempt, *Li* lithium, *Plac* placebo, *Cit* citalopram, *Quet* quetiapine, *VLP* valproate, *LTG* lamotrigine, *CBZ* carbamazepine

**Table 2:** Studies focusing on suicide and mortality, long-term follow-up of lithium-treated patients and epidemiological studies including record linkage

Year/country	Author	Diagnoses	Medication/type of study	Number of patients	Duration	Results
1972/UK	Barraclough	Affective disorders	Retrospective, clinical survey	100 suicide cases		>1/5 of these 100 suicides might have been prevented if lithium had been used
1977/USA	Fieve	Affective disorders	Li	20	78 weeks	0 suicide
			Retrospective cohort study			
1984/Czech Republic	Hanus and Zapletalek	Affective disorders	Li treatment compared to non-Li treatment/retrospective cohort study	95	5 years	4 SA during Li therapy
					20 % reduction	25 SA during non-Li therapy
1991/UK	Coppen et al.	Affective disorders	Li	103	11-year follow-up	10 deaths from other causes, expected number 18.31; no suicides
			Retrospective cohort study			
1991/Denmark	Vestergaard and Aargaard	Affective disorders	Li	133	5 years	No advantage
			Retrospective cohort study			
1992/Germany, Denmark, Canada, Austria	Müller-Oerlinghausen et al. (a)	Affective disorders	Li	827	Ø 81 months = 5600 patient-years	44 deaths, total number of deaths was not different from what would be expected in a matched sample of the general population
			Retrospective cohort study	IGSLI cohort		
1992/Germany	Müller-Oerlinghausen et al. (b)	Affective disorders	Comparison of Li treatment with non-Li treatment period	68	8 years, at least 12 months of Li treatment	2 suicides, 4 SA during Li treatment (in 55 patients)
						4 suicides, 7 SA when not Li-treated
			Retrospective cohort study			
1994/Germany	Felber and Kyber	Affective disorders	Comparison of Li treatment with non-Li treatment period	36	14 years, 6.8 years with lithium, 7.2 years without lithium	64 SA during non-Li treatment,
						7 SA during Li treatment, reduction SA 10:1, reduction suicides 3:1
			Retrospective cohort study			
1995/Sweden	Nilsson	Affective disorders	Comparison of Li treatment with non-Li treatment period	362	At least 1 year of Li treatment	129 deaths, risk to die was 1.7 times higher when not Li-treated and 4.8 times higher to commit suicide when not Li-treated
			Retrospective cohort study			
1996/Germany, Canada, Denmark, Austria, Sweden	Wolf et al.	Affective disorders	Li	1056	Average length of Li treatment 86.2 months	Mortality of lithium-treated patients did not differ from general population
			Retrospective cohort study	Total treatment period 90.982 months		
				IGSLI cohort		
1998/Italy	Bocchetta et al.	Affective disorders	Li	100	Ø 9.6 years observation period	10 suicides—9 of them after discontinuation of Li therapy, suicide risk 24-fold higher when not Li-treated
			Retrospective cohort study			
1999/USA	Baldessarini et al.	Bipolar disorder	Li	310	Ø 6.36 years Li, of that group, 128 patients with Ø 3.7 years without lithium + 8.2 years observation period before lithium therapy	7-fold lower rate of suicidal events during Li therapy compared to time before Li therapy rate of suicidal events 20-fold higher after discontinuation
			Retrospective cohort study			
2000/Sweden	Kallner et al.	Affective disorders	Li	497	30 years	80 % higher suicide rate when Li therapy was discontinued
			Retrospective cohort study			
2000/Denmark	Brodersen et al.	Affective disorders	Li	133	2 years observation period + follow-up after 16 years	40 deaths, including 11 suicides, mortality was twice that of general population (due to suicides), mortality from all other causes was similar; suicides occurred more often in those not compliant with therapy
			Retrospective cohort study			
2000/UK	Coppen et al.	Unipolar Bipolar Schizoaffective	Retrospective cohort study	67 unipolar, 30 bipolar, 6 schizoaffective	1977–1995	24 deaths, 21 deaths from natural causes, 2 suicides, 1 road accident, overall suicide rate was 1.3 per 1000 patient-years of observation
						Expected suicide rate per 1000 years in this population between 5.4 and 10.2
2001/Germany	Conell et al.	Affective disorders	Comparison of Li treatment with non-Li treatment period	33	Ø 10.3 years without lithium	Anti-suicidal effect did not differ after restarting the Li therapy
					Ø 19.8 years with lithium	
						Response rate after discontinuation and restarting lower
			Retrospective cohort study			
2001/Germany (region Saxony)	Fülle et al.	All diagnoses	Analysis of suicide cases within all hospitals in Saxony compared to a matched control group	800 patients including 400 suicides + 400 controls		Control group had 6 times more often lithium long-term treatment compared to suicide group
						Lithium therapy was under-represented within the suicide group
2001/Germany, Denmark, Austria	Ahrens et al.	Affective disorders	Li	167	Ø 6.7 years	Reduction of SA in excellent responders (3 SA), moderate responders (14 SA), and poor responders (21 SA)
			Retrospective cohort study	Subgroup of IGSLI cohort	1120 treatment years	
			Comparison of response rates			
2001/USA	Coryell et al.	Affective disorders	Retrospective cohort study	15 suicides compared to 15 non-suicides		6 of suicides and 8 of controls were thought to take lithium, 9 of SA and 8 of controls were on lithium
				41 suicide attempts compared to 41 non-SA		
2003/USA	Goodwin et al.	Bipolar disorder	Li	20.623	At least one prescription with Li or VLP or CBZ	Lithium-treated patients had 1.5- to 3-fold reduced suicide risk compared to VLP-treated patients
			VLP	Data from health insurances		
			CBZ			
			Retrospective cohort study			
2005/Denmark	Kessing et al.		Li	13.186	Patients with at least one lithium prescription compared to patients who never received lithium	Lithium patients had 0.44-fold reduced suicide rate
			Retrospective cohort study	Data from a national register		
2005/Italy	Bocchetta et al.	Affective disorders	Li	1394 patients	5474 years of lithium treatment	Patients treated >5 years with lithium had reduced mortality rate (like general population)
			Retrospective cohort study	18.154 patient years		
2005/Switzerland	Angst et al.	Affective disorders	Li	406 patients	40 years	45 suicides, Li-treated patients had lower suicide rate which did not differ from general population
			Prospective epidemiological study			
2006/Spain	Gonzales-Pinto et al.	Bipolar I	Li	72	10 years	5.2-fold reduced risk for suicidal behavior or SA in patients with stable and good Li response
			Prospective cohort study			
2008/USA	Collins and McFarland	Bipolar disorder	Li	12662 patients		Overall 12 suicides, 81 SA, 2 suicides, 15 SA (Li)
			VLP	2558 (Li)		
			Other anti-convulsive medication	2214 (VLP)		2 suicides, 41 SA (VLP)
				2002 (gabapentin)		7 suicides, 19 SA (gabapentin)
				242 (CBZ)		
						4 SA (CBZ)
						Lowest (0.78) suicide rate per 1000 person years in Li-treated patients
2009/Australia	Keks et al.	Bipolar disorder	Retrospective cohort study	35 suicides		Only 4 of them were treated with lithium
2011/Germany	Neuner et al.	All diagnoses	Analysis of all suicides within psychiatric hospitals compared to a matched control group without suicides	133 hospital suicides vs. 133 non-suicide patients		Affective disorders: 59 suicide patients (0 patients with lithium) 60 control patients (12 patients with lithium)

*SA* suicide attempt, *Li* lithium, *Plac* placebo, *Cit* citalopram, *Quet* quetiapine, *VLP* valproate, *LTG* lamotrigine, *CBZ* carbamazepine

**Table 3:** Studies on the potential anti-suicidal effects of lithium as a trace element in drinking water

Year	Author	Measurement	Number of samples	Results
2009/Japan	Ohgami et al.	Li level in drinking water	18 municipalities	Standardized mortality ratio (SMR) negatively correlated with Li levels
2011/UK	Kabacs et al.	Li level in drinking (tap) water	47 samples from 47 subdivisions	No association between lithium levels in drinking (tap) water and mortality from suicide in the East of England
2011/Austria	Kapusta et al.	Li level in drinking water	6460 lithium measures of 99 Austrian districts	Suicide rate, SMR inversely associated with Li levels
2013/Greece	Giotakus et al.	Li level in drinking water	149 water samples from 34 prefectures	Tendency for lower suicide rates in the prefectures with high levels of lithium in drinking water
2013/USA	Blüml et al.	Li level in public water	3123 lithium water samples, 226 counties	Higher lithium levels in the public drinking water were associated with lower suicide rates
2015/Italy	Vita et al.	Li level in drinking water	Review	Higher levels in drinking water may be associated with reduced risk of suicide in the general population

*Li* lithium

**Table 4:** Reviews and meta-analyses

Year/country	Author	Diagnoses	Medication	Number of patients	Duration	Results
1997/Italy, USA	Tondo et al.	Affective disorders	Comparison of Li treatment with non-Li treatment period	Over 17,000		8.6-fold higher risk for suicide and SA within the non-Li-treated group
						After discontinuation of Li therapy, 7-fold increase of suicidal events
			Retrospective cohort study			
2005/UK	Cipriani et al.	Affective disorders	Li	1389 Li-treated patients	Long-term treatment	Li reduced suicide risk and all-cause mortality by approximately 60 %
			Other compounds			
				2069 with other-substances-treated patients		
2006/US	Baldessarini et al.	Affective disorders (mainly unipolar)	Li	33,340 patients	85.229 patient years	Lithium-treated patients had 5-fold reduced risk for suicides and SA compared to patients without lithium
2007/USA, Italy	Guzzetta et al.	Unipolar depression	Li	328	4.65 years with lithium	Overall risk for suicides and SA was 88.5 % lower with lithium
					6.27 years without lithium	
2013/UK	Cipriani et al.	Mood disorders	15 comparisons	6674 participants	48 randomized controlled trials	Li more effective than placebo in reducing number of suicides and deaths from any causes

*SA* suicide attempt, *Li* lithium

#### Randomized controlled trials—not specifically focusing on suicidality or mortality

Three early trials of patients with affective disorders comparing lithium with placebo and imipramine reported zero deaths due to suicide in the treatment groups with a small number of suicides in the placebo groups (Coppen et al. 1971; Prien et al. 1973a, b).

In 1989, Dorus et al. published a double-blind, placebo-controlled trial of depressed and non-depressed alcoholics. One death from all causes occurred in the placebo group (*n* = 89) and no deaths were reported in the lithium group (*n* = 82). The comparative efficacy of lithium and amitriptyline in the maintenance treatment of recurrent unipolar depression was investigated by Greil et al. (1996) in a RCT with 81 patients over a treatment period of 2.5 years. This study has the longest follow-up period to date. One suicide occurred in the amitriptyline group (*n* = 41), and no suicides were reported in the lithium group (*n* = 40). Another publication from this group (Greil et al. 1997a) assigned patients with bipolar disorder to lithium or carbamazepine. No suicides were observed in the lithium group (*n* = 74), while one suicide and one attempted suicide occurred in the carbamazepine group (*n* = 70) over a 2.5-year follow-up period. Furthermore, the efficacy of carbamazepine compared to lithium was examined in a subgroup of 90 patients with schizoaffective disorder (Greil et al. 1997b). Similarly, no suicides or suicide attempts were reported in the lithium group (*n* = 43), while four suicide attempts were reported in the carbamazepine group (*n* = 47) over the treatment period of 2.5 years.

In a sample of 30 patients with refractory major depression participating in a randomized double-blind, placebo-controlled trial to lithium or placebo, over a time period of 4 months, Bauer et al. (2000) reported one suicide in the placebo group and no suicides in the lithium group. A study of 49 elderly patients with depression followed for 2 years reported four patient deaths (two on placebo and two on lithium) from causes unrelated to treatment (Wilkinson et al. 2002)

A placebo-controlled trial of lamotrigine and lithium maintenance treatment in patients with bipolar I disorder revealed no suicides in the lithium group (*n* = 121) and one suicide in the lamotrigine group (*n* = 221) over the treatment period of 18 months (Calabrese et al. 2003). A double-blind 18-month trial (Findling et al. 2005) of lithium versus divalproex maintenance treatment in pediatric bipolar disorder found no significant differences in suicide or suicide attempts between groups (*n* = 60).

In contradiction, one suicide has been reported in the lithium treatment group within a double-blind period and one suicide in an open-label phase of a 12-month randomized double-blind controlled clinical trial comparing olanzapine versus lithium in the maintenance treatment of bipolar disorder (Tohen et al. 2005).

A study Kok et al. 2007 comparing the effect of lithium augmentation with phenelzine among patients with treatment-resistant major depressive disorder reported two deaths in the lithium group (*n* = 15) while using nortriptyline. In the phenelzine group (*n* = 14), one patient died taking phenelzine who took lithium a few months after finishing the trial and two other patients died while continuing use of phenelzine.

The first placebo-controlled randomized trial designed specifically to investigate the influence of lithium on suicidal behavior was published in 2008 by Lauterbach et al. Survival analyses indicated no significant difference in suicidal acts between lithium- and placebo-treated individuals (adjusted hazard ratio 0.517; 95 % CI 0.18–1.43); further, post hoc analyses revealed that all completed suicides had occurred in the placebo group accounting for a significant difference in incidence rates (*P* = 0.049). Within a treatment period of 1 year, no suicides occurred within the lithium group (*n* = 84) whereas three suicides occurred within the placebo group (*n* = 83). No significant differences in suicide attempts between the groups were found with seven suicide attempts occurring in both the placebo and lithium group.

A large randomized open-label trial by Geddes et al. 2010 comparing lithium plus valproate therapy versus monotherapy for relapse prevention in patients with bipolar I disorder documented two patient deaths from all causes in the lithium group (*n* = 110) and three patient deaths from all causes in the valproate group (*n* = 110).

Another study demonstrating contradictory findings reported one suicide in a lithium treatment group from an open randomized effectiveness study comparing lamotrigine versus lithium as maintenance treatment in bipolar I disorder. One suicide attempt was also reported in the lamotrigine group (*n* = 77) (Licht et al. 2010).

Oquendo et al. published a study in 2011 investigating the effect of lithium compared to valproate in the prevention of suicidal behavior in patients with bipolar disorder over a period of 2.5 years. No suicides were reported over the follow-up. Six suicide attempts were reported in the lithium group (*n* = 49) and eight suicide attempts in the valproate group (*n* = 49). A large study by Weisler et al. 2011 comparing the continuation of quetiapine versus switching to placebo or lithium for the maintenance treatment in 2.438 patients with bipolar I disorder showed one suicidal/accidental gunshot wound during the open-label treatment with quetiapine. Khan et al. (2011) published a study primarily designed to investigate whether the anti-suicidal effects of lithium can be prospectively evaluated using lithium as an augmenting agent to antidepressants. A subgroup of the patients assigned to citalopram and lithium achieved therapeutic serum levels and had significantly higher Sheehan Suicidality Tracking Scale (S-STS) remission rates compared to patients assigned to citalopram and placebo groups. The authors hypothesized that lithium when used in therapeutic doses may augment a direct therapeutic effect of citalopram on suicidal thoughts and behaviors.

#### Studies focusing on suicidality and mortality: long-term follow-up of lithium-treated patients—epidemiological studies including record linkage

One of the first descriptions of lithium’s anti-suicidal properties is dated back to 1972 when Barraclough described the current and past clinical history of 100 suicide cases. He postulated that as many as a fifth of these suicides may have been prevented if lithium had been used.

In 1977, Fieve reported that in 20 patients with lithium long-term treatment (78 weeks), no suicidal acts were observed demonstrating evidence for the anti-suicidal effects of this drug.

Hanus and Zapletálek (1984) came to similar conclusions when they analyzed data from 95 patients who were treated with lithium for approximately 5 years and compared suicide attempt rates during this treatment time to a past time without lithium therapy. They observed a 20 % reduction in suicide attempts.

In 1991, Coppen et al. published a paper in which the authors analyzed the mortality of 103 patients attending a specialized lithium clinic. Only ten patients died during the study due to causes unrelated to treatment. Interestingly, the expected number of deaths due to suicide in this sample was 18.31, and considering no deaths from suicide were observed, this suggests that lithium reverses the excess mortality associated with recurrent mood disorders, including that from suicide. However, another study showed no advantage of lithium in terms of the overall mortality in 133 patients with affective disorders followed for 5 years while taking lithium (Vestergaard and Aagaard 1991).

Müller-Oerlinghausen 1992a, together with the International Group for The Study of Lithium Treated Patients (IGSLI), demonstrated in a large international study comprising 827 lithium-treated patients with affective disorders that their mortality did not differ compared to a matched healthy population. Epidemiological studies indicate a 2- to 3-fold higher standardized mortality in untreated bipolar patients compared to the general population.

The Berlin group also studied the occurrence of suicides and suicide attempts in 68 patients with affective disorders and a history of suicide attempt while on and off lithium treatment. They observed only one suicide in patients with regular lithium intake. Eleven of 13 patients showed suicidal or parasuicidal behavior after lithium discontinuation which was proposed as an indication of lithium’s anti-suicidal effect independent of the general episode suppressing effect (Müller-Oerlinghausen et al. 1992b).

In 1994, Felber and Kyber found a 10 to 1 reduction of suicide attempts and a 3 to 1 reduction in the number of suicides in patients taking lithium compared to an untreated time.

A Swedish study of 362 patients with affective disorders found that the relative risk of suicide was 4.8 times higher when patients were off lithium compared to taking lithium (Nilsson 1995), which is in line with the findings from the IGSLI study.

Using three different methods to calculate the standardized mortality ratio (SMR), Wolf et al. (1996) reported that the mortality of lithium-treated patients with affective disorders (*n* = 1056) is not significantly different from that of the general population.

A similar finding was shown by Bocchetta et al. in 1998. Lithium treatment in 100 patients with affective disorders was associated with a 6-fold decreased incidence of suicide attempts. During the observational period, ten suicides occurred, while nine deaths occurred when patients had discontinued lithium therapy. The risk of suicide was 24 times higher when patients went off lithium.

The Boston group (Baldessarini et al. 1999) demonstrated that the incidence of suicide increased by 20 times in the first year after ending lithium therapy in 300 bipolar patients. Interestingly, they also had found that the suicide risk was reduced by 6.5 times during lithium treatment.

A Swedish study published in 2000 by Kallner et al. showed a mortality-decreasing effect of lithium in 497 patients with affective disorders who were treated in a specialized outpatient lithium clinic. The suicide rate was increased by 80 times when patients stopped taking lithium and discontinued the specialized treatment.

In contradiction to these latter studies, the mortality of 133 Danish lithium-treated patients with affective disorders was not decreased compared to the general population (Brodersen et al. 2000). Specifically, 11 suicides were observed within the lithium group; however, it is of interest that the authors observed a significantly increased suicide risk in patients who were not compliant compared to those who took lithium regularly.

Coppen (2000) described a 75 % reduction in suicide rate in a group of 103 patients on lithium maintenance therapy recruited into a mood disorder clinic in 1977. By 1995, 24 patients had died. The SMR was calculated using the age-specific death rates for England and Wales for the midpoint of the period of observation. The expected number of deaths was 25.89, giving an SMR of 0.93. The expected number of suicide deaths was less than one. The overall suicide rate was 1.3 per 1000 patient years of observation. Patients in this study were selected because of severity and high recurrence rate, where higher rate of suicides was expected.

Conell et al. (2001) investigated a group of 33 patients with affective disorders who went through periods of discontinuation of their lithium treatment. Although the response rates were lower after restarting lithium, the anti-suicidal effect showed no significant differences between times when patients were on and off their lithium. This result could be interpreted as evidence that lithium’s anti-suicidal properties are not dependent on its mood-stabilizing effects.

Using a different approach to investigate the anti-suicidal effects of lithium, Fülle et al. (2001) collected treatment information from all suicides within hospitals in Saxony (Germany). Lithium medication was less common in the patients committing suicide compared to patients not attempting suicide. Similarly, Neuner et al. (2011) compared 133 clinic suicides with 133 non-suicide controls and found that none of the patients in the suicide group were treated with lithium while 12 patients in the control group were on lithium therapy.

The anti-suicidal effect of lithium has even been documented across different lithium response categories. For example, Ahrens and Müller-Oerlinghausen (2001) found a reduction in suicide attempts not only in the excellent lithium responders but also among patients with a moderate to poor response to lithium. This adds further evidence that lithium may possess a suicide protective effect in addition to its mood-stabilizing properties.

Another study showed no difference in completed suicide between lithium and non-lithium-treated patients (Coryell et al. 2001). Six (40.0 %) of the lithium-treated patients who committed suicide and eight (53.3 %) of the control patients were thought to have been taking lithium in the preceding week.

In 2003, Goodwin et al. reported on a large sample of 20,623 health-insured patients with bipolar disorder. Patients who had received lithium had a 1.5- to 3-fold reduced risk of suicide or suicide attempts compared to patients receiving valproate.

Kessing et al. 2005 used data from a national register to analyze 13,186 patients who had received one or more prescriptions for lithium and compared these to patients who had never had a prescription for lithium. The authors found that patients with more than one lithium-prescription had a 0.44-fold reduced suicide rate compared to those who had only one lithium prescription. Bocchetta (2005) conducted a naturalistic study of 1394 patients with affective disorders and reported a reduced mortality rate in patients treated with lithium for more than 5 years.

Findings from the Zurich cohort, which followed 406 patients with affective disorders for over 40 years (Angst et al. 2005), reported a lower mortality rate among patients treated with lithium. The mortality rate among the lithium-treated patients was not different from the mortality rate in the general population. Gonzales-Pinto et al. 2006 reported on 72 long-term (10-year) observations of patients with bipolar I disorder. This study found a significantly lower (5.2-fold) occurrence of suicide attempts and suicidal behavior in lithium-treated patients.

Collins and McFarland (2008) investigated 12,662 Medicaid patients and demonstrated that lithium-treated bipolar patients had the lowest number of suicide attempts compared to patients treated with other mood stabilizers.

Keks et al. 2009 investigated 35 bipolar patients who had committed suicide, where four of these patients were lithium-treated and more than one third had never received lithium.

#### Studies on potential anti-suicidal effects of lithium as a trace element in drinking water

Another approach to determine lithium’s suicide protective effects is to investigate the ecological association between suicide rates and lithium in drinking water. Four studies compared lithium levels in drinking water to suicide rates in different countries and regions. Two studies (Ohgami et al. 2009; Kapusta et al. 2011) one from Japan and one from Austria concluded that areas with higher lithium levels in the drinking water had lower suicide rates.

Kabacs et al. (2011) measured lithium levels in tap water in 47 subdivisions of the East of England and correlated these levels of suicide standardized mortality ratio in each subdivision. The author’s findings did not support an association between lithium level in drinking water and suicide rates. Giotakos et al. (2013), however, reported lower suicide rates in prefectures with high levels of lithium in drinking water. Blüml et al. (2013) also showed that lithium levels were negatively associated with suicide rates using 3123 state-wide lithium measurements of the public water supply of Texas. A recently published review (Vita et al. 2015) of the association between lithium in drinking water and suicide rates supports the association between higher levels of lithium in drinking water and a reduced risk of suicide in the general population.

#### Reviews and meta-analyses

One of the first reviews examining suicide and suicide attempts in patients with affective disorders was published by Tondo et al. in 1997. The authors demonstrated in a pooled sample of more than 17,000 that suicide risk was decreased by 8.6 times in patients treated with lithium compared to patients not taking lithium.

Another meta-analysis conducted by Cipriani et al. (2005) including more than 3000 patients demonstrates again a lower level of suicide or suicidal events and a reduced overall mortality among lithium-treated patients compared to patients treated with other medications. The same group from Oxford (Cipriani et al. 2013) recently published an updated systematic review and meta-analysis of randomized controlled trials showing that lithium is an effective treatment for reducing suicide risk in patients with affective disorders.

Baldessarini et al. (2006) conducted a meta-analysis of 45 studies including more than 33,000 patients showing that patients treated with lithium had a 5-fold reduced risk of suicide or suicide attempts compared to patients treated with other substances.

When analyzing data of 328 Sardinian patients with unipolar depression, Guzzetta et al. (2007) found a significant lower risk for suicide attempts and suicides in lithium-treated patients.

### Discussion

Patients with mood disorders have a 30-fold greater risk of suicide compared to the general population. On September 4, 2014, the World Health Organization (WHO) published the first report on suicide prevention (WHO 2014). It summarizes that more than 800,000 people die by suicide every year, almost one person every 40 s. It also states that more research is urgently needed in the field of effective therapeutic strategies. One pharmacological strategy is the long-term lithium treatment of patients with affective disorders irrespective of whether they are in the bipolar, unipolar, or schizoaffective subgroups.

Compelling evidence provides strong support that lithium has a suicide protective effect over the long-term course in patients with mood disorders. Lithium-treated patients have a reduced risk of suicide as well as a reduced risk of mortality from other causes when compared with other drugs (e.g., valproate) and placebo.

Strong cause and effect evidence comes from RCTs investigating the mood-stabilizing effect of lithium. While complimentary evidence is given by careful analysis of cohorts of well-documented patients receiving lithium over many years under constant direct monitoring, little is known about the underlying mechanism behind the association between lithium and a reduced suicide risk. Two possible mechanisms of the preventive effect of lithium have been suggested. First, lithium is a mood-stabilizing medication. Therefore, patients with a good response to lithium typically have no further or at least less severe/less frequent affective episodes which should in turn decrease the risk of suicidal behavior. Second, it is suggested that lithium might decrease aggression and impulsivity. Studies in humans and animals have shown the association between lithium use and lower levels of aggression and impulsivity which could also lead to a reduction of suicidal behavior (Comai et al. 2012).

Neurobiological research has focused on lithium’s influence on neurotransmitters such as serotonin, noradrenalin, and dopamine, on the cortisol stress hormone system, the γ-aminobutyric acid, second messenger systems such as the inositol metabolism, glycogen synthase kinase 3, and more. The most common hypothesis is that lithium leads to a decrease in impulsivity and aggression via several influences within the nerve cell (Müller-Oerlinghausen and Lewitzka 2010; Mühlbauer and Müller-Oerlinghausen 1985). Over-activity of the corticotrophin-releasing hormone as well as dysfunction of the noradrenergic and serotonergic systems may be implicated in suicide (Steiner et al. 2008; Erhardt et al. 2013). Further, these dysfunctions may be linked to microglial hyperactivity, and chinolidinic acid deriving from tryptophan could lead to a lowered cerebral level of tryptophan and serotonin (Steiner et al. 2012). It could be that lithium, through its serotonin agonistic properties, counteracts this deficiency at the neurotransmitter level.

Another approach to study the underlying mechanisms of the anti-suicidal effects of lithium is through animal models. Ohmura et al. **(**2012) investigated whether major mood-stabilizing drugs used for the treatment of bipolar disorder could suppress impulsive-like action in a three-choice serial reaction time task in rats. The authors suggested that lithium might suppress impulsive behavior and in turn decrease the risk of suicide. This suggests that lithium could be a beneficial treatment for impulsivity-related disorders.

Further research is necessary to identify causal pathways in the association between lithium use and a decreased risk of suicide. It is still unclear what specific psychological effects arise from lithium use that may in turn reduce suicidal behavior. It would also be of importance to determine if there is a threshold or a specific level of lithium required for its anti-suicidal properties to come into effect and whether lithium is effective in reducing suicidal behavior in other psychiatric diseases. A few studies from the 1960s and 1970s examined lithium’s effect on a broad range of psychiatric problems including brain-related abnormal behavior, mental retardation with abnormal behavior, children with disruptive and aggressive behavior, and prisoners with high levels of aggressiveness and impulsiveness. Future research should determine lithium’s effect on other psychiatric diseases associated with a high risk of suicide such as borderline personality disorders or substance use disorders.

Despite the large number of studies showing evidence of lithium’s anti-suicidal effects, there are some limitations of these studies that are worth noting. Several studies investigated a selective group of patients as good adherence is a prerequisite of lithium treatment. These selective patients may possess protective factors such as better coping with long-term treatment or treatment in specialized settings while other patients (with worse adherence) may not. Lithium’s anti-suicidal properties are less clear in these less selective patients. As discussed earlier, suicide research is methodologically and ethically challenging, therefore only a small number of randomized controlled trials exist. However, there are observational studies showing that discontinuation of lithium medication increases the risk of suicide risk considerably, even after long episodes of treatment. It is unlikely that this phenomenon is a withdrawal effect, similar to what is typically observed when antidepressant drugs are discontinued.

#### Considerations for clinicians

Patients with affective disorders have a 30 times greater risk of suicide compared to the general population. According to several international guidelines, lithium is still a first-line option for acute and maintenance treatment of affective disorders (Lam et al. 2009). It is also an augmentation strategy in the therapy of depressive disorders (Bschor et al. 2007). The practical use of lithium requires pharmacological competence in the individual prescribing. Patients need to be comprehensively informed, and several medical examinations should be done before starting treatment with lithium. Once lithium therapy has started, constant monitoring of lithium level and blood parameters is essential.

As with any other medication, lithium can cause adverse effects. The most frequent acute effects are as follows: hand tremor, increased thirst, nausea, diarrhea, and abdominal distress. Most of these effects typically disappear after several weeks of initiation of treatment or lose intensity. A number of other long-term adverse effects can occur including weight gain, edema, euthyroid goiter, hypothyroidism, hyperparathyroidism with hypocalcemia, acne/psoriasis, and hair loss. Kidney damage is a relevant problem in the long-term treatment with lithium (McKnight et al. 2012; Müller-Oerlinghausen et al. 2012), and lithium intoxication can be a serious complication. The well-known symptoms and signs of acute or chronic intoxication should be known to the prescriber as well as to the patient. Patients showing any of these signs require immediate emergency medical care. Hemodialysis may be needed in the case of acute or chronic lithium intoxication depending on the level and the status of the patient. The clear benefit of lithium long-term medication outweighs the adverse effects, and these effects can be mitigated with proper care and monitoring. Patients who are unable or unwilling to be monitored continuously should not be given lithium.

Overall, the clinical experience of physicians who work with lithium, especially in specialized departments, shows that lithium can be handled safely.

There are some known clinical predictors (Grof et al. 1993) of good lithium response (Table 5). For example, it is well known that lithium is especially effective in patients with a typical course including full remission between episodes, only mood congruent psychotic symptoms, and no psychiatric comorbidity. There tends to be fewer efficacies in patients with bipolar disorder with an atypical course including residual symptoms between episodes, mood-incongruent psychotic features, and high psychiatric comorbidity.

**Table 5:** Predictors of good lithium response

Positive family history for bipolar disorder	
Previous remission with lithium	
Classical: euphoric manic episodes	
Full remission between episodes	
Good adherence	

In light of these clinical indicators of lithium response, we would typically recommend the use of lithium in patients with a good lithium response; however, if a patient shows partial response, while exhibiting suicidal behavior, or is at high-risk for suicidal behavior, the continuation of lithium therapy should be considered. Otherwise, owing to safety concerns, we would not recommend the continuation of long-term lithium medication among patients whose response is uncertain or poor. Physicians should be especially cautious when planning to discontinue lithium therapy because of the lack of mood-stabilizing effect. If the patient is burdened with a considerable suicide risk, and based on their genetic load or individual history, the continuation of lithium could be debated (from the anti-suicidal perspective); however, this kind of use is off-label and therefore needs to have a documented explanation.

#### Ongoing research

A recent multicenter, double-blind, placebo-controlled trial investigating how fast the anti-suicidal effect of lithium appears is underway (EudraCT Number: 2013-000970-31; ClinicalTrials.gov identifier number NCT02039479). Data that currently exist are mostly from patients with affective disorders with long-term treatments. To date, no study has investigated the time lithium takes to exert its anti-suicidal effect. In this study, adults with a diagnosis of major depression (uni- and bipolar) with moderate to severe suicidal thoughts/behavior (measured with the Sheehan Suicidality Tracking Scale (S-STS) and the Columbia Suicide Severity Rating Scale (C-SSRS)) will be allocated to treatment as usual and lithium or placebo. Following randomization, treatment will be administered for 5 weeks and change in the S-STS (primary outcome) will be measured weekly by the study physician as well as daily by the patients. In addition to the S-STS, secondary outcomes include change in depressive, anxiety, and impulsivity symptoms as well as number of psychotherapy sessions and other medications received. The results of this study should inform whether the anti-suicidal effect of lithium evokes early at the beginning of the treatment.

## Conclusions

Lithium is an effective treatment for reducing the risk for suicide and suicide attempts in patients with affective disorders over the long-term course. Data also suggest that the expected higher overall mortality in patients with mood disorders using lithium is decreased.
